# Punctate Palmoplantar Keratoderma Type 1 With a Multigenerational Family History: A Case Report

**DOI:** 10.7759/cureus.116184

**Published:** 2026-09-13

**Authors:** Fernando J Medina-Olivares, Claudia Fernanda Patiño Orellana, Felix R Hernández Altamirano, Elisa Vega-Memije

**Affiliations:** 1 Department of Dermatology, Hospital General "Dr. Manuel Gea González", Mexico City, MEX; 2 Department of Dermatopathology, Hospital General "Dr. Manuel Gea González", Mexico City, MEX

**Keywords:** aagab, buschke-fischer-brauer disease, genodermatosis, palmoplantar keratoderma, punctate palmoplantar keratoderma

## Abstract

Punctate palmoplantar keratoderma type 1 (PPKP1), also known as Buschke-Fischer-Brauer disease, is a rare inherited keratinization disorder characterized by multiple hyperkeratotic papules on the palms and soles. It is typically inherited in an autosomal dominant pattern with variable penetrance and has been associated with pathogenic variants in the *AAGAB* gene. We report the case of an 86-year-old man with an asymptomatic palmoplantar dermatosis of more than 50 years' duration who was evaluated during hospitalization for an unrelated medical condition. Examination revealed numerous yellowish-brown punctate and confluent keratotic papules and plaques involving the palms and soles, with marked hyperkeratosis over pressure-bearing areas. Similar palmoplantar lesions were reported in two of his sons and one sister, clinically suggesting an autosomal dominant inheritance pattern. Histopathologic examination of a plantar lesion demonstrated well-demarcated compact orthokeratotic hyperkeratosis overlying an epidermal depression, with irregular acanthosis and no significant dermal inflammation. The clinical presentation, multigenerational family history, and histopathologic findings supported a diagnosis of probable PPKP1. Molecular testing for *AAGAB* variants was not performed; therefore, the diagnosis was clinically and histopathologically supported rather than genetically confirmed. This case highlights the characteristic clinicopathologic features of probable PPKP1 after more than five decades of evolution and underscores the diagnostic value of a detailed family history in inherited palmoplantar keratodermas.

## Introduction

Palmoplantar keratodermas (PPKs) comprise a heterogeneous group of inherited and acquired disorders characterized by abnormal thickening of the stratum corneum of the palms and soles. Inherited PPKs can be clinically classified according to their distribution as diffuse, focal, or punctate [1].

Punctate palmoplantar keratoderma type 1 (PPKP1), also known as Buschke-Fischer-Brauer disease, is a rare genodermatosis characterized by the progressive development of multiple hyperkeratotic papules on the palms and soles. The disorder is generally inherited in an autosomal dominant pattern with variable penetrance and has been associated primarily with heterozygous loss-of-function variants in the *AAGAB* gene [2,3]. Molecular testing is required to confirm pathogenic variants and may be particularly useful when phenotypic overlap with other punctate PPKs creates diagnostic uncertainty.

Clinical expression is variable, and lesions may become more prominent with increasing age and repeated mechanical stress. Histopathologic findings are supportive but not pathognomonic, making clinicopathologic correlation and family history particularly important in establishing the diagnosis [1,4].

We present the case of an 86-year-old man with more than five decades of PPK and similar findings in multiple family members. This case illustrates how probable PPKP1 can be clinically and histopathologically recognized in the absence of molecular confirmation through the integration of lesion morphology, anatomic distribution, histopathologic findings, and multigenerational family history. Accordingly, the diagnosis in this patient is clinically and histopathologically supported rather than genetically confirmed.

## Case presentation

An 86-year-old man hospitalized in the Internal Medicine Department for abdominal sepsis was referred to Dermatology for the evaluation of asymptomatic chronic palmoplantar dermatosis. The exact age at onset could not be established; however, the patient reported that the lesions had been present for more than 50 years and had gradually become more prominent over time. Before hospitalization, he had not received specific medical treatment for the palmoplantar lesions and managed the rough hyperkeratotic areas by periodically filing them.

Dermatologic examination revealed a bilateral and symmetric dermatosis involving the palms and soles, with extension to the fingertips, lateral aspects of the digits, and periungual regions. Numerous discrete yellowish-brown punctate keratotic papules were observed, several showing a central depression. Some lesions coalesced into larger, irregular hyperkeratotic plaques with a rough surface. Associated xerosis and superficial fissuring were also present. Plantar involvement was considerably more pronounced, particularly over pressure-bearing areas, where confluent hyperkeratotic lesions produced a cobblestone-like appearance (Figure 1). Nail examination showed onychodystrophy and distal subungual hyperkeratosis.

**Figure 1:**
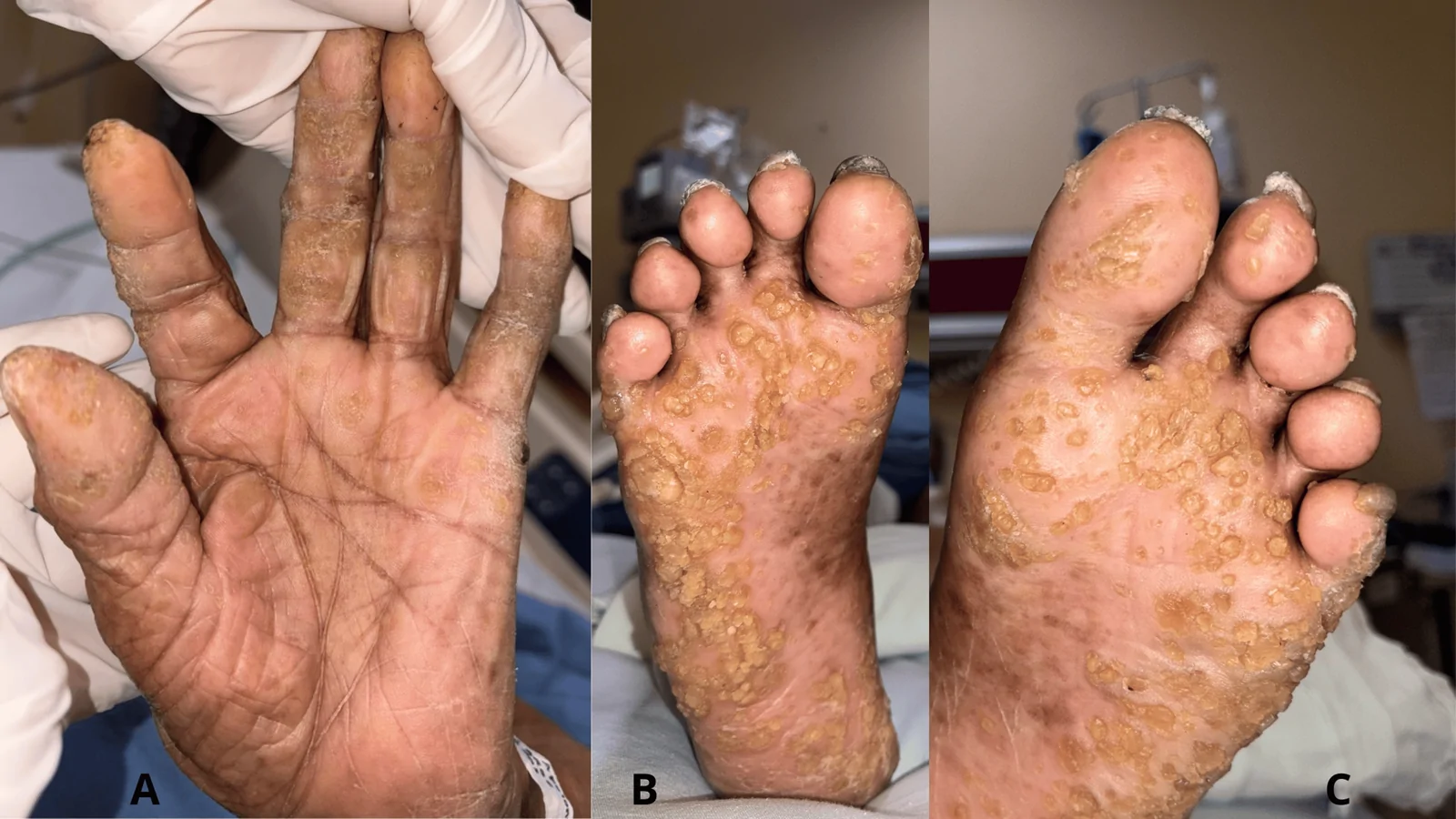
Clinical features of probable punctate palmoplantar keratoderma type 1 (A) Multiple discrete yellowish-brown punctate keratotic papules involving the palmar surface, fingertips, and lateral aspects of the digits, with areas of confluent hyperkeratosis. (B) Bilateral and symmetric plantar involvement with numerous punctate and confluent keratotic papules and plaques, predominantly affecting pressure-bearing areas. (C) Closer view of the plantar surface demonstrating multiple round hyperkeratotic papules, several with central depressions, together with confluent thick keratotic plaques producing a cobblestone-like appearance.

A detailed family history revealed similar palmoplantar lesions in two of the patient's sons and one sister. Additional information regarding the exact age at onset, clinical progression, and severity of the lesions in the affected relatives was not available. No genetic testing was performed in the patient or his affected relatives.

An incisional 4-mm punch biopsy was obtained from a keratotic lesion on the plantar surface of the right foot. Histopathologic examination demonstrated a well-demarcated area of marked compact orthokeratotic hyperkeratosis overlying an epidermal depression, accompanied by irregular acanthosis. The underlying dermis showed no significant inflammatory infiltrate or other relevant abnormalities (Figure 2).

**Figure 2:**
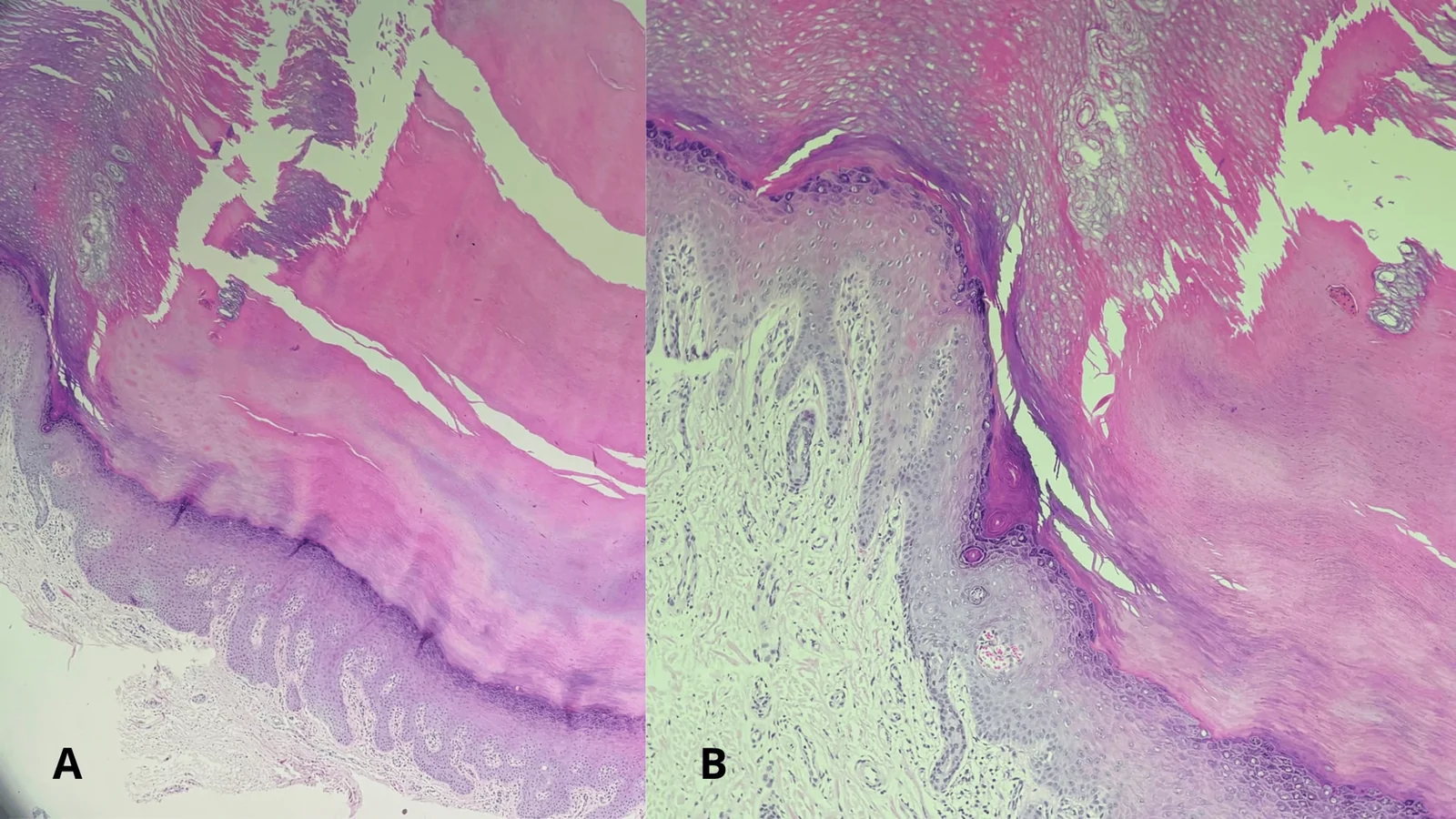
Histopathologic features of a plantar keratotic lesion (A) Low-power view showing marked, well-demarcated compact orthokeratotic hyperkeratosis overlying an epidermal depression, with associated irregular acanthosis (hematoxylin and eosin stain, ×4). (B) Higher-power view highlighting compact orthokeratotic hyperkeratosis over the epidermal depression and the absence of a significant inflammatory infiltrate in the underlying dermis (hematoxylin and eosin stain, ×20).

The long-standing bilateral and symmetric distribution, together with the multigenerational family history, made multiple palmoplantar verrucae less likely. Histopathologically, the absence of a cornoid lamella favored PPKP1 over punctate porokeratosis, while the lack of papillomatosis and viral cytopathic changes argued against verrucae.

Based on the characteristic punctate palmoplantar morphology, bilateral and symmetric distribution, predominance of confluent hyperkeratosis in pressure-bearing areas, prolonged clinical course, multigenerational family history, and supportive histopathologic findings, a clinically and histopathologically supported diagnosis of probable PPKP1 (Buschke-Fischer-Brauer disease) was made.

Given the absence of symptoms, advanced age, and concurrent systemic illness, an individualized conservative approach was selected, consisting of topical 40% urea cream twice daily, frequent emollient use, and measures to reduce friction and repetitive mechanical trauma. The patient died the day after the skin biopsy during the same hospitalization; therefore, dermatologic follow-up and assessment of treatment response were not possible. Genetic evaluation had been suggested because of the strong family history, but molecular testing could not be performed. A patient perspective was not obtained before the patient's death and therefore could not be included. Informed consent for publication of the clinical information and images was obtained from the patient's legally authorized representative.

## Discussion

PPKs constitute a clinically and genetically heterogeneous group of disorders characterized by excessive keratinization of the palms and soles. Inherited forms are traditionally categorized according to their clinical distribution as diffuse, focal, or punctate, although considerable phenotypic overlap exists [1].

PPKP1 is an uncommon inherited PPK characterized by multiple discrete hyperkeratotic papules involving the palms and soles. It follows an autosomal dominant inheritance pattern with variable penetrance. Lesions typically begin during adolescence or early adulthood and progressively increase in number and size with age [4,5]. Individual papules may remain discrete or coalesce into thick hyperkeratotic plaques, particularly in areas exposed to pressure, friction, or repetitive trauma [4].

The present patient exhibited a particularly extensive phenotype after more than five decades of evolution, with numerous punctate lesions and confluent keratotic plaques involving pressure-bearing plantar surfaces. Similar lesions in two sons and one sister were clinically suggestive of an autosomal dominant inheritance pattern; however, this inheritance pattern was not genetically established. Neither the patient nor his affected relatives underwent molecular testing for *AAGAB* or other genes associated with punctate PPKs.

Previous molecular studies have shown that PPKP1 is frequently associated with heterozygous loss-of-function variants in *AAGAB*, located on chromosome 15q23 [2,3,6,7]. *AAGAB* encodes the alpha- and gamma-adaptin-binding protein p34, which is involved in intracellular receptor trafficking and regulation of keratinocyte proliferation. Experimental studies have suggested that loss of *AAGAB* function may alter epidermal growth factor receptor trafficking and increase growth factor signaling, thereby contributing to abnormal keratinocyte proliferation [2,3]. These molecular mechanisms were not directly assessed in the present patient. Considerable inter- and intrafamilial phenotypic variability has been reported among individuals carrying *AAGAB* variants, suggesting that additional modifying factors may influence clinical expression [6].

Histopathologic findings in PPKP1 are supportive but not pathognomonic. Characteristic features include a well-defined epidermal depression associated with prominent compact orthokeratosis, frequently accompanied by hypergranulosis and variable acanthosis, with little or no dermal inflammation [1,3,4]. In the present case, the biopsy demonstrated compact orthokeratotic hyperkeratosis overlying an epidermal depression, with irregular acanthosis and no significant inflammatory infiltrate, supporting the clinical diagnosis.

The differential diagnosis includes punctate porokeratosis, multiple palmoplantar warts, spiny keratoderma, and acrokeratoelastoidosis. Palmoplantar warts may interrupt dermatoglyphics and exhibit thrombosed or ectatic capillaries clinically; histopathologic findings include papillomatosis, koilocytosis, and prominent capillaries. Punctate porokeratosis is distinguished histologically by a cornoid lamella overlying an epidermal depression with reduction or absence of the granular layer. Acrokeratoelastoidosis preferentially affects the margins of the hands and feet and is characterized histologically by fragmentation and reduction of elastic fibers [1,4]. Recognition of the distribution of lesions, family history, and histopathologic pattern therefore helps distinguish PPKP1 from these mimickers.

Additional reports have highlighted the considerable phenotypic variability of PPKP1. Intrafamilial differences in disease severity associated with aging have been described, and some genetically confirmed cases may present predominantly or exclusively with plantar lesions [8,9]. At the molecular level, heterozygous *AAGAB* variants have also been associated with increased growth factor signaling, providing further evidence that altered receptor trafficking and keratinocyte proliferation contribute to disease pathogenesis [10]. Although an association between PPKP1 and malignancy has historically been proposed, a recent systematic review did not confirm an increased malignancy risk and found insufficient evidence to recommend specific cancer surveillance solely on the basis of PPKP1 [11].

Management of PPKP1 should be individualized according to symptom burden, extent of disease, functional impairment, patient age, comorbidities, and treatment goals. Topical emollients and keratolytic agents, including urea, salicylic acid, and lactic acid, may be used to reduce hyperkeratosis and associated fissuring or discomfort. Systemic retinoids may be considered in selected patients with extensive, symptomatic, or functionally limiting disease, although treatment-related adverse effects and recurrence after discontinuation should be taken into account [1,5,12,13]. In the present patient, the absence of symptoms, advanced age, and significant concurrent systemic illness favored an individualized conservative approach with topical 40% urea, emollients, and measures to reduce mechanical trauma.

A limitation of this report is the absence of molecular testing in the patient and affected family members. Therefore, the presumed autosomal dominant inheritance pattern could not be genetically confirmed, and phenotypically overlapping punctate PPKs cannot be definitively excluded. Nevertheless, the characteristic morphology and distribution of the lesions, their prolonged course, multigenerational familial clustering, and supportive histopathologic findings provide strong clinicopathologic support for probable PPKP1.

This case also illustrates that inherited keratinization disorders may remain undiagnosed for decades when lesions are asymptomatic or regarded as a benign familial trait. Obtaining a detailed family history can therefore provide a critical diagnostic clue in patients presenting with chronic palmoplantar hyperkeratosis.

## Conclusions

PPKP1 should be considered in patients with multiple punctate hyperkeratotic lesions of the palms and soles, particularly when lesions are bilateral, symmetric, become confluent over pressure-bearing areas, and occur in multiple family members. Although histopathologic findings are not pathognomonic, focal compact orthokeratotic hyperkeratosis over epidermal depressions can provide important supportive evidence when interpreted in the appropriate clinical context.

This case demonstrates a marked phenotype clinically and histopathologically compatible with probable PPKP1 after more than five decades of evolution and emphasizes the diagnostic value of integrating morphology, distribution, histopathologic findings, and multigenerational family history. However, molecular testing is required to confirm pathogenic gene variants and is particularly important for definitive genetic characterization and counseling when clinically indicated.

## References

[1] Thomas BR, O'Toole EA (2020). Diagnosis and management of inherited palmoplantar keratodermas. Acta Derm Venereol.

[2] Giehl KA, Eckstein GN, Pasternack SM (2012). Nonsense mutations in AAGAB cause punctate palmoplantar keratoderma type Buschke-Fischer-Brauer. Am J Hum Genet.

[3] Pohler E, Mamai O, Hirst J (2012). Haploinsufficiency for AAGAB causes clinically heterogeneous forms of punctate palmoplantar keratoderma. Nat Genet.

[4] Endara-Camacho A, Novelo-Soto A, Manríquez-Robles A, Toussaint-Caire S, Vega-Memije ME (2019). Queratodermia palmoplantar punctata de Buschke-Fisher-Brauer. Dermatol Rev Mex.

[5] Nomura T, Yoneta A, Pohler E (2015). Punctate palmoplantar keratoderma type 1: a novel AAGAB mutation and efficacy of etretinate. Acta Derm Venereol.

[6] Giehl KA, Herzinger T, Wolff H (2016). Eight novel mutations confirm the role of AAGAB in punctate palmoplantar keratoderma type 1 (Buschke-Fischer-Brauer) and show broad phenotypic variability. Acta Derm Venereol.

[7] Pohler E, Huber M, Boonen SE (2014). New and recurrent AAGAB mutations in punctate palmoplantar keratoderma. Br J Dermatol.

[8] Kono M, Fukai K, Shimizu N (2017). Punctate palmoplantar keratoderma type 1 with a novel AAGAB frameshift mutation: intrafamilial phenotype variation due to ageing. J Eur Acad Dermatol Venereol.

[9] Hasegawa A, Hayashi R, Shimomura Y, Hirashima M, Abe R (2021). Only plantar lesion of punctate palmoplantar keratoderma with a novel missense mutation in the AAGAB gene: two Japanese familial case reports and review of reported mutations. J Dermatol.

[10] Pöhler E, Zamiri M, Harkins CP (2013). Heterozygous mutations in AAGAB cause type 1 punctate palmoplantar keratoderma with evidence for increased growth factor signaling. J Invest Dermatol.

[11] Gram SB, Bjerrelund J, Jelsig AM, Bygum A, Leboeuf-Yde C, Ousager LB (2023). Is punctate palmoplantar keratoderma type 1 associated with malignancy? A systematic review of the literature. Orphanet J Rare Dis.

[12] Nomura T, Moriuchi R, Takeda M (2015). Low-dose etretinate shows promise in management of punctate palmoplantar keratoderma type 1: case report and review of the published work. J Dermatol.

[13] Bodemer C, Steijlen P, Mazereeuw-Hautier J, O'Toole EA (2021). Treatment of hereditary palmoplantar keratoderma: a review by analysis of the literature. Br J Dermatol.

